# Post-transplant colitis after kidney transplantation: clinical, endoscopic and histological features

**DOI:** 10.18632/aging.202345

**Published:** 2020-12-22

**Authors:** Rossella Gioco, Lidia Puzzo, Marco Patanè, Daniela Corona, Giuseppe Trama, Pierfrancesco Veroux, Massimiliano Veroux

**Affiliations:** 1 General Surgery Unit, University Hospital of Catania, Catania 95123, Italy; 2 Pathology Unit, Department of Medical and Surgical Sciences and Advanced Technologies, University of Catania, Catania 95123, Italy; 3 Organ Transplant Unit, University Hospital of Catania, Catania 95123, Italy; 4 Department of Biomedical and Biotechnological Sciences, University of Catania, Catania 95123, Italy; 5 Gastroenterology Unit, University Hospital of Catania, Catania 95123, Italy

**Keywords:** kidney transplantation, cytomegalovirus, colitis, inflammatory bowel disease, colonoscopy

## Abstract

Chronic immunosuppression may increase the risk of post-transplant infection and medication-related injury and may also be responsible for the increased risk of gastrointestinal complications in kidney transplant recipients. Differentiating the various forms of post-transplant colitis is challenging, since most have similar clinical and histological features. This study evaluated the incidence of post-transplant gastrointestinal complications during screening colonoscopy. Kidney transplant recipients undergoing a colonoscopy for any reasons in the period 2014-2018 were included. Among the 134 patients completing the colonoscopy, 74 patients (56%) had an abnormal finding: an adenoma was found in 25 patients (18.6%), while 19 patients (14.1%) had colitis. Mycophenolic acid/related colitis was the most common colitis (6%), while 7 patients (5.2%) developed a *de novo* inflammatory bowel disease. Patients with post-transplant colitis were younger and with shorter time from transplant compared to patients without colitis. In conclusions, immunosuppression may predispose kidney transplant recipients to an increased risk of post-transplant colitis. Diagnostic colonoscopy should be encouraged in all transplant patients with refractory diarrhea and gastrointestinal symptoms to allow a prompt diagnosis and a timely treatment, finally improving the quality of life and long-term outcomes of affected patients.

## INTRODUCTION

Kidney transplantation is considered the gold standard treatment in patients affected by end-stage renal disease, since it significantly improves the quality of life and patient survival compared to dialysis [1]. The chronic use of immunosuppressive therapy to contrast the risk of acute rejection, exposes kidney transplant recipients to a variety of long-term complications, including the gastrointestinal complications, which are a common cause of morbidity and mortality after kidney transplantation [2–4].

Gastrointestinal complications in kidney transplant recipients may be a consequence of viral infections, such as those from Cytomegalovirus (CMV) [5–8], and of the chronic injury of immunosuppression to the gastrointestinal mucosa. The use of Mycophenolate mofetil (MMF) and mycophenolic acid (MPA), which are commonly used in the immunosuppression maintenance after transplantation, has been largely associated with an increased risk of gastrointestinal complications in kidney transplant recipients [2–4, 9].

Post-transplant inflammatory bowel disease (IBD) may arise from an inappropriate immune response to intestinal antigens, resulting in a continuous intestinal inflammation [2]. In kidney transplant recipients the immunosuppressive therapy, which could theoretically contrast this inflammatory process, may paradoxically allow for dysregulation of the intestinal immune system, finally resulting in the development of post-transplant *de novo* IBD, despite being immunosuppressed [2, 10–13].

Clinical and histological features of gastrointestinal complications and IBDs in kidney transplant recipients have been rarely reported. In this study, we evaluated the incidence, the endoscopic and the histological features of gastrointestinal complications in a population of kidney transplant recipients undergoing screening colonoscopy.

## RESULTS

A total of 134 patients completed the colonoscopy and were included in the study (Table 1). There was a slight prevalence of male sex (56.7%), with a mean age of 55 ± 10 years, and mean time from transplantation to colonoscopy of 9.8 ± 9.6 years: moreover, most patients (85%) received a tacrolimus- and mycophenolic acid-based immunosuppression. Glomerulonephritis was the most common cause of ESRD (59.7%), followed by autosomal dominant polycystic disease (26.7%). Diarrhea (44.7%) and gastrointestinal bleeding or anemia (18.6%) were the most common indications for colonoscopy in symptomatic patients, while colonoscopy was performed for colorectal cancer screening in 39 (29.2%) transplant patients.

**Table 1:** Clinical and endoscopic features of study population (n=134).

	**n(%)**
**Age (yrs)**	55 ± 10
**Sex (M/F)**	76/58
**BMI (Kg/m^2^)**	26.6 ± 5.18
**Waiting list**	19.5 ± 24
**Pre-transplant dialysis**	40.1 ± 38.3
**Cause of ESRD**	
Glomerulonephritis	80 (59.7)
ADPKD	36 (26.7)
Diabetes	9 (6.8)
Other	9 (6.8)
**Donor Age (yrs)**	47 ± 17.5
**Immunosuppression (n)**	
Induction	40 (29.8)
Tac+MPA+ Ster	114 (85)
CyA+MPA+Ster	10 (7.5%)
Ever+Tac+Ster	10 (7.5%)
**Indications for colonoscopy**	
Diarrhea	60 (44.7)
Screening	39 (29.2)
Gastrointestinal Bleeding	25 (18.6)
Abdominal Pain	10 (7.5)
**FIT**	
Positive	96 (71.6)
Negative	38 (28.4)
**FCP positive**	
Positive	114 (85)
Negative	30 (15)
**Colonoscopic findings**	
Normal	59 (44.0)
Adenoma/Polyps	25 (18.6)
Diverticulosis	20 (14.9)
Ulcer/erosion	14 (10.5)
Hyperemia	5 (3.7)
Other (haemorrhoids, colon ischemia, pseudomembranous colitis)	11 (8.2)

**Legend: BMI:** body mass index; **ESRD**: end-stage renal disease; **ADPKD:** autosomal dominant polycystic kidney disease; **TAC:** tacrolimus; **MPA**: mycophenolic acid; **Ster:** steroids; **CyA:** cyclosporin; **Ever:** Everolimus; **FIT:** faecal immunochemical test; **FCP:** Faecal calprotectin.

A normal colonoscopy was found in 59 (44%) patients, while 74 patients (56%) had an abnormal finding: an adenoma was found in 25 patients (18.6%), while diverticulosis was diagnosed in 20 patients (14.9%). In 19 patients (14.1%) the colonoscopic findings were suggestive of post-transplant colitis (hyperemia, erosion and/or ulcers) [15]. FIT was positive in 85% of the study group: in patients with normal colonoscopy findings, FIT was positive in 33 of 59 patients (55.9%, negative predictive value 63.1%), while in patients with positive colonoscopy, FIT was positive in 61 of 75 patients (81.3%, positive predictive value 63.5%). The overall sensibility and specificity of FIT in kidney transplant population was 81.3% and 40.6%, respectively. Mean FCP value in patients with positive colonoscopy (1366± 748.4 mcg/g) was not significantly different to that observed in patients with normal colonoscopy (1104.5± 686.4 mcg/g, p=0.203), suggesting that FCP could not be used for screening of gastrointestinal disease in kidney transplant recipients.

There was no significant prevalence of female sex between transplant patients with normal colonoscopy and positive patients, but patients with post-transplant colitis were significantly younger than patients with negative colonoscopy and, above all, than patients with non-colitis positive colonoscopic findings (51.5±11.6 *vs* 58.6±13.2 years, p=0.02) (Tables 2, 3). Moreover, time from transplant was significantly shorter in patients with post-transplant colitis compared with patients with negative colonoscopy (7.1±4.3 *vs* 9.4±3.1, p=0.048). While most patients with negative colonoscopy were asymptomatic, almost all patients with positive colonoscopy presented diarrhea and/or rectal bleeding.

**Table 2:** Clinical characteristics of patients with normal colonoscopic findings compared with patients with positive colonoscopy.

**Characteristic**	**Normal colonoscopy (n=59)**	**Positive colonoscopy –colitis (n=19)**	**P-Value**
**Female Sex(n,%)**	29 (49.1)	8 (42.1)	0.673
**Age(years, mean)**	54.8±12.1	51.5±11.6	0.072
**Time from transplant (years)**	9.4	7.1	**0.048**
**FOBT positive (%)**	59.3	79	0.784
**FCP (mcg/g, mean)**	1047.2	1609.1	0.071
**Diarrhea (n, %)**	10 (16.9)	19 (100)	**< 0.01**
**Rectal bleeding (n,%)**	3 (5)	1 (5.2)	.824
**Serum Creatinine (mg/dl, mean)**	1.5	1.4	.776

Legend: FIT: faecal immunochemical test; FCP: Faecal calprotectin.

**Table 3:** Clinical characteristics of patients with colitis compared with patients with positive colonoscopy but without colitis.

**Characteristic**	**Positive colonoscopy –colitis (n=19)**	**Positive colonoscopy non-colitis (n=56)**	**P-value**
**Female Sex(n,%)**	8 (42.1)	21 (37.5)	0.362
**Age(years, mean)**	51.5±11.6	58.6±13.2	**0.02**
**Time from transplant (years)**	7.1	10.7	0.327
**FOBT positive (%)**	79	80	0.824
**FCP (mcg/g, mean)**	1609.1	1423	0.430
**Diarrhea (n, %)**	19 (100)	8 (14.2)	**< 0.01**
**Rectal bleeding (n,%)**	1 (5.2)	20 (35.7)	**< 0.05**
**Serum Creatinine (mg/dl, mean)**	1.4	1.6	.811

Legend: FIT: faecal immunochemical test; FCP: Faecal calprotectin.

### Histological findings

Clinical diagnoses were classified according to final histologic examination (Table 4). Twelve patients (8.9%) had an adenoma with low- or high-grade dysplasia: among them, 2 patients underwent a colonic resection. Two patients had a colorectal cancer: one had a complete colonoscopic resection, while the other patient presented with an advanced disease and died two months after the diagnosis.

**Table 4:** Histological diagnoses (n=43).

**Diagnosis**	**N(%)**
**Hyperplastic polyp**	9 (20.9)
**Adenoma with low-grade dysplasia**	8 (18.6)
**Adenoma with high-grade dysplasia**	4 (9.3)
**Colo-rectal cancer**	2 (4.6)
**MPA-colitis**	8 (18.6)
**Crohn disease**	4 (9.3)
**Ulcerative colitis**	3 (9.9)
**CMV colitis**	4 (9.3)
**Ischemic colitis**	2 (4.3)

**Figure 1:**
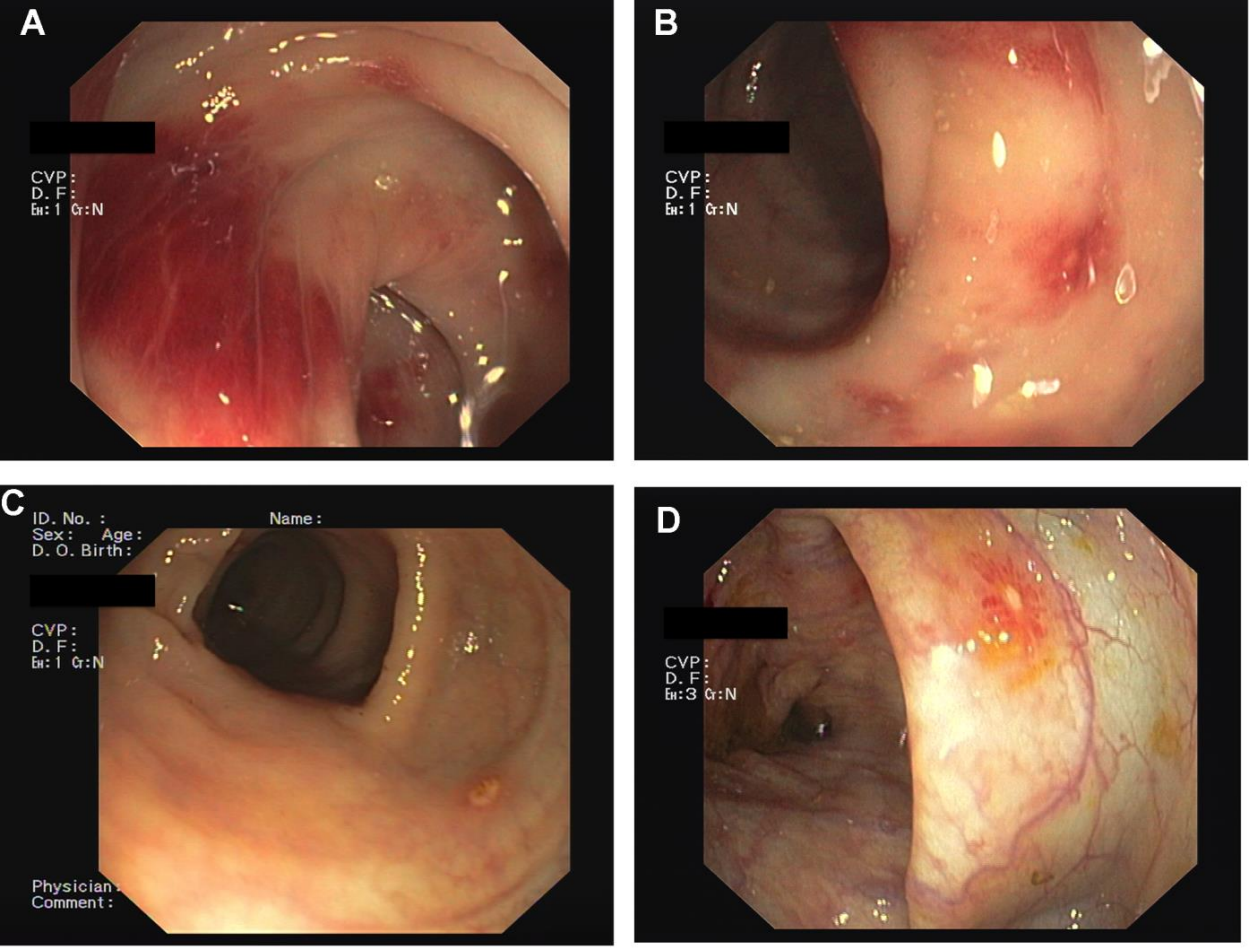
**Mycophenolic Acid colitis.** Endoscopic Findings. Colonoscopy demonstrated the presence of severe hyperemia to the right colon (**A**, **B**), with erosion of the colonic mucosa (**C**, **D**).

**Figure 2:**
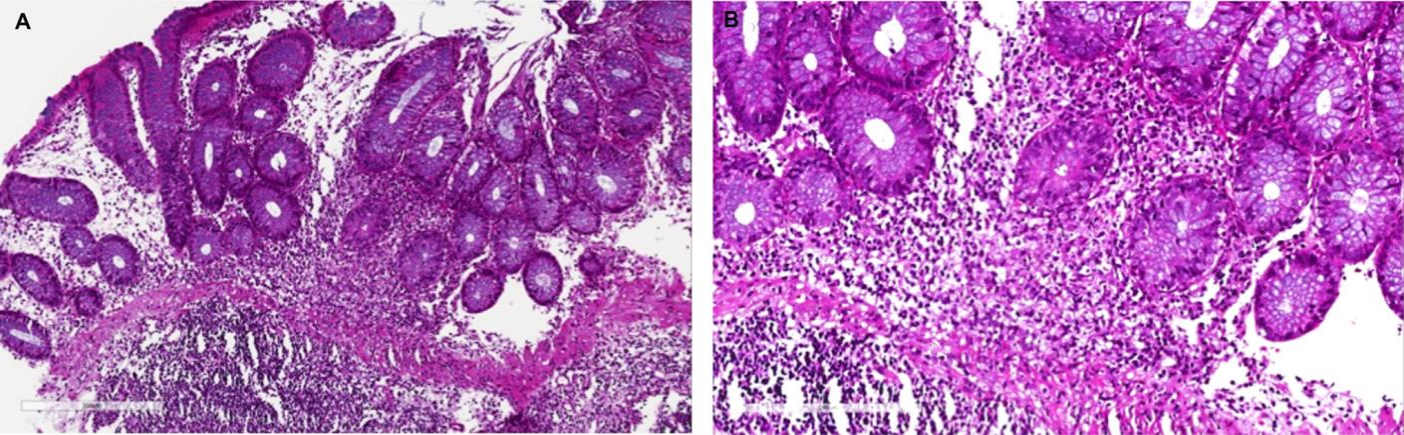
**Mycophenolic acid colitis.** Histological evaluation at 10x (**A**) and 20x (**B**). Colon biopsy retrieved from right colon during diagnostic colonoscopy shows (**A**) a severe eosinophils and plasma cells infiltrate of the submucosal layer (magnification 10x), (**B**) confirmed at higher magnification (20x).

**Figure 3:**
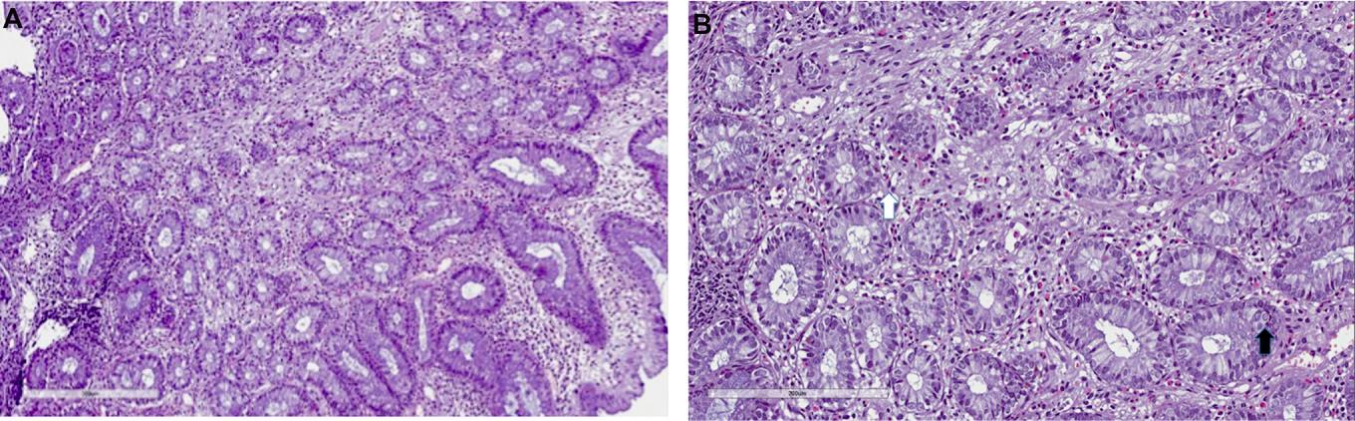
**Mycophenolate mofetil colitis.** Histological examination. Biopsy of right colon: (**A**) severe eosinophils, lymphocytes and plasma cells infiltrate (10x), with (**B**) severe cryptitis (20x, white arrow) and cell apoptosis (black arrow).

Nineteen patients (14.1%) developed a post-transplant colitis (Table 4). Eight patients (6%) presented a MPA/colitis: mean age was 55.1± 11.3, female patients were 50%, and all patients presented with diarrhea. The mean time from transplant to MPA-related diarrhea onset was 84.7±34.3 months. All patients but one had positive FIT, and mean FCP was 1716±675.3 mcg/g. Colonoscopic findings included erosions, colonic ulcers and hyperemia (Figure 1). Histological findings revealed a mild to severe eosinophils and plasma cells infiltrate with cryptic abscesses (Figures 2, 3). All patients had a reduction of MPA dosage: at mean follow up of 31±9.7 months, 5 patients had a complete resolution of diarrhea after MPA reduction, while 3 patients had only a mild improvement. In patients with clinical resolution mean FCP was 320±121 mcg/g compared to patients without complete resolution who exhibited a mean FCP of 745±223.4 mcg/g (p = 0.065).

**Figure 4:**
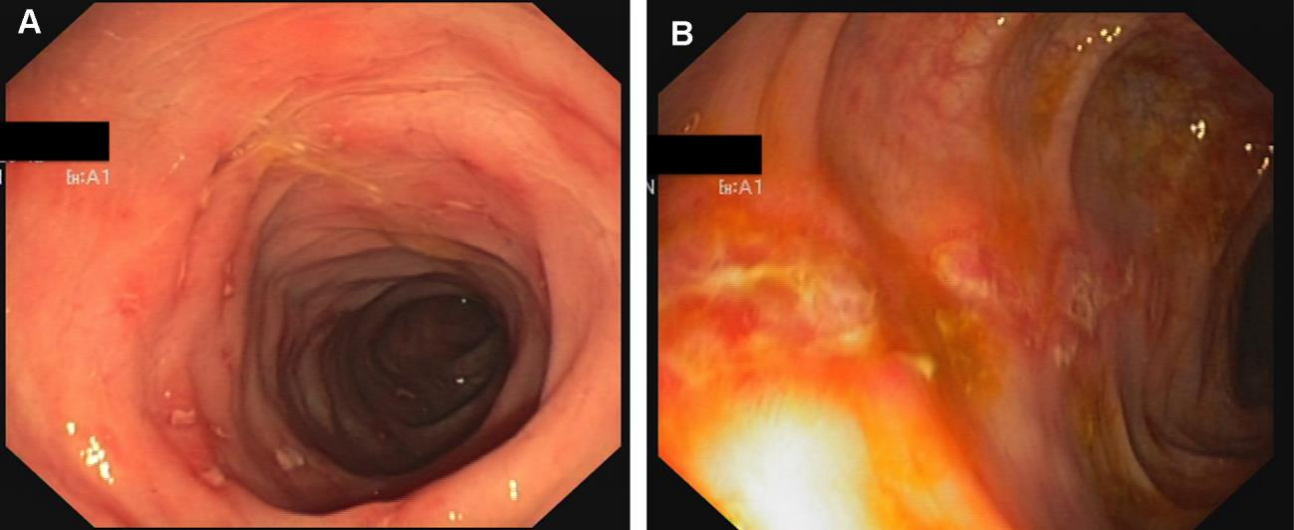
**Crohn-like colitis.** Endoscopic findings. Multiple erosions and hyperemia of the mucosa of rectum (**A**) and sigma (**B**).

**Figure 5:**
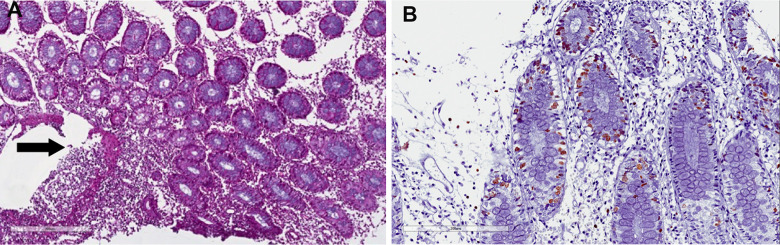
**Crohn-like colitis.** Histological features. (**A**) Irregular, severe eosinophils, neutrophils and plasma cells infiltrate in the submucosal layer (arrow). (**B**) Proliferation index Ki67 in the crypt, without apoptosis. Elevated Ki67 suggests regenerative activity as a consequence of crypt injury (cryptitis).

Seven patients (5.2%) developed a *de novo* IBD (3 ulcerative colitis and 4 Crohn disease). All patients with ulcerative colitis (UC) were men with a mean age of 46 years and developed UC after a mean time of 59 months after transplantation, suggesting that chronic exposure to immunosuppression could have a role in the development of IBD. In all patients, UC presented with diarrhea (> 7 stool/day), abdominal pain and, in one patient, with rectal bleeding. Colonoscopy revealed the presence of sigmoid and rectal ulcers with pancolitis (Figure 4). Histological examination demonstrated the presence of diffuse colitis with lymphoplasmatocitory infiltrate (Figure 5). One patient developed a sigmoid stenosis requiring a sigmoidectomy. Sigmoid stenosis recurred two years after surgical intervention, but the patient remained asymptomatic and in clinical remission. The other two patients are in clinical remission at a mean follow up of 28 months.

Four patients developed a Crohn disease (CD). There was an equal distribution between male and female patients with a mean age of 46.2 years. As observed in patients with ulcerative colitis, CD developed late after transplantation, after a mean time from transplant of 123 months.

Two patients required a surgical intervention: one female patient with refractory disease underwent an ileal resection, while a male patient underwent a surgical procedure of perianal fistulectomy. The other two patients are in clinical remission at a mean follow up of 32 months.

**Figure 6:**
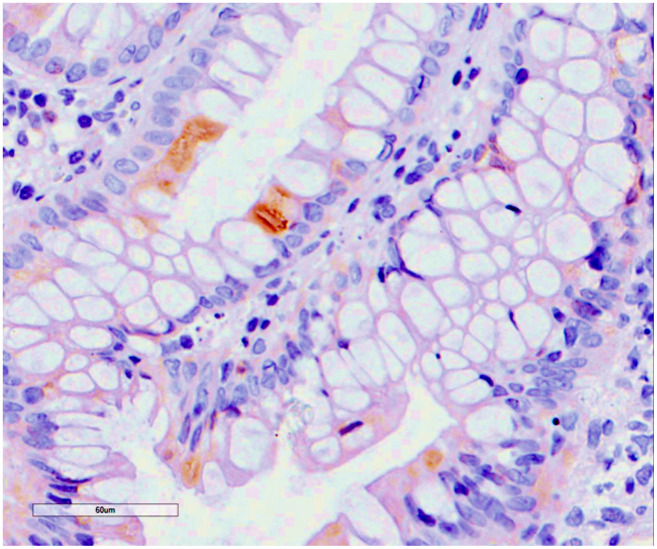
**Cytomegalovirus colitis.** Histological findings. Positivity for early cytomegalovirus antigen on immunochemistry.

Finally, four patients (2.9%) developed a CMV colitis (Figure 6). There was an equal number of men and women with a mean age of 51.5 years. All patients had PCR positivity for serum CMV DNA and complained of diarrhea and abdominal pain. CMV-colitis developed early after transplantation, after a mean time of 8.8 months, which is the period of highest incidence of CMV infection in kidney transplant recipients.

Interestingly, CMV colitis was associated with MPA-colitis in one patient and with *C. difficile* infection in another patients, suggesting that CMV colitis is linked to a higher level of immunosuppression during the early period after transplantation. Indeed, the patient who developed the CMV colitis together with *C. difficile* infection, developed an acute rejection in the early post-transplant period, which required a higher dose of immunosuppression. This resulted in CMV colitis and intestinal perforation, for which the patient underwent a colonic resection. Unfortunately, the patient died 14 days after surgical procedure due to multiple organ failure.

## DISCUSSION

Gastrointestinal complications may occur in up to 80% of renal transplant patients, usually as a consequence of the chronic use of immunosuppression [14, 15]. Diarrhea and abdominal pain are the most commonly reported gastrointestinal complaints, and they may be associated with a lower quality of life and in an increased risk of poor graft outcome and patient survival [16, 17].

Post-transplant colitis represents a challenging complication of kidney transplantation, since there is no consensus on clinical and histological features, due to the likely overlap between similar colitis, such as MMF colitis and graft-*vs–*hos*t* colitis, and to the non-specific clinical symptoms of post-transplant colitis. Moreover, most studies investigated only a small proportion of symptomatic patients undergoing colonoscopy and this could underestimate the true prevalence of such diseases.

This study reported one of the largest series of diagnostic colonoscopies in kidney transplant recipients, investigating the incidence of gastrointestinal complications and post-transplant colitis. Diarrhea and rectal bleeding were the most common indications for colonoscopy and a positive colonoscopy was found in 56% of patients. Adenoma and diverticulosis were the most common findings among patients with positive colonoscopy, while the incidence of post-transplant colitis was 14.1%, but it rose up to 25.6% considering only patients with positive colonoscopy.

Fecal immunochemical test and fecal calprotectin are widely applied stool biomarkers for the diagnosis of gastrointestinal disease: FIT is an accurate screening test for colorectal cancer in general population and could avoid colonoscopy in 75–80% of non-transplant patients [18, 19], and emerging data suggest its potential role in monitoring disease activity in patients with IBD [20, 21]. However, in kidney transplant population FIT showed a low sensitivity and a reasonable specificity [22, 23], and its role in the diagnosis and monitoring of gastrointestinal complications in kidney transplant population has rarely been investigated. In our study, FIT was positive in 85% of patients, with an overall sensibility and specificity of 81.3% and 40.6%, respectively, suggesting that FIT may not efficaciously discriminate those patients requiring a colonoscopy for gastrointestinal complications.

Fecal calprotectin is a marker for intestinal inflammation, and has been extensively evaluated as a biomarker for predicting inflammation in patients with IBD [21]. A recent study demonstrated that the combined use of FIT and FCP could be useful for diagnosing an endoscopically active disease [21]. In solid organ transplant setting, FCP was used for monitoring the acute rejection of intestinal transplantation, and showed a good sensitivity but low specificity for the diagnosis of intestinal rejection [24].

However, the role of FCP in kidney transplant recipients has been rarely evaluated. Although patients with colitis had high levels of FCP, our study failed to find a significant correlation between FCP values and disease activity: there was no difference in FCP values between patients with positive and negative colonoscopic findings and also between patients with colitis and patients with positive colonoscopy without colitis; moreover, patients who were in clinical remission did not have a significant decrease in FCP values. The high incidence of abnormal levels of FCP in kidney transplant recipients could be probably related to the chronic not-specific inflammation of the colonic mucosa caused by immunosuppression.

Taken together, these data suggest that both FIT and FCP could not be useful for diagnosis and monitoring of the disease activity of post-transplant colitis in kidney transplant recipients.

MMF/MPA colitis was the most common post-transplant colitis, affecting the 42.1% of patients with positive colonoscopy. In a recent report, Pittmann et al. [2], described the clinical and histological features of 51 kidney transplant recipients who underwent a colonoscopy for gastrointestinal complaints. Most patients presented with diarrhea and anemia, and 12 patients (23.5%) were ultimately considered to have a MMF-related injury. Calmet et al. [25] in a colonoscopic study on 397 solid organ transplant recipients, found a prevalence of MMF-colitis of 9%, and this incidence was particularly higher in kidney transplant recipients, where MMF-colitis was reported in 23% of patients.

Endoscopic and histological features of MMF/MPA/colitis are not specific and may be similar to those of IBD, graft-*vs*-host disease and ischemic colitis [3, 30]. Common histological findings are extensive atrophy of the crypt with mild to severe eosinophil or plasma cells infiltrate [2, 3, 9, 26, 27].

This was confirmed in our study, where MPA/colitis presented with erosions and hyperemia at colonoscopy, with a severe eosinophils and plasma cells infiltrate at histological examination. Notably, no cryptic abscesses were identified. The treatment of MMF colitis includes a reduction or, in more severe forms, complete discontinuation of MPA [9, 26, 27]: in our study, a reduction of MPA dosage resulted in a complete resolution or in a significant improvement in 41.2% of patients, while in most patients it resulted only in a mild improvement of symptoms.

Seven patients (5.2%) developed a *de novo* IBD. *De novo* IBDs are an uncommon complication of kidney transplantation, with only 46 cases reported in a recent review [26]. *De novo* IBDs usually present late after transplantation, as observed in our experience, suggesting a potential etiopathogenetic role of chronic exposure of intestinal mucosa to immunosuppressive therapy [26]. Clinical, endoscopic and histological features resemble those observed in general population but, in kidney transplant recipients, *de novo* IBDs could have a more aggressive course than that in general population [31–36], due to the likely interaction between immunosuppression and IBD-specific therapy [26, 28–33]. This assumption was confirmed in our study, where almost half of patients required a surgical intervention due to IBD-related complications.

Mesalazine and cortico-steroids are the mainstays of treatment of *de novo* IBD even in transplant patients [26, 28, 33], although recent reports suggested the use of anti-TNFα antibodies for patients refractory to standard therapy [31, 33]: however, approximately 20% of patients are refractory to therapy and ultimately need surgical treatment with colectomy [31, 33].

CMV is the virus that most commonly infects patients undergoing solid organ transplantation, with significant consequences on graft and patient survival, and 50-70% of patients experience significant viral replication and symptomatic illness, resulting in a tissue-invasive disease [34, 35]. The gastrointestinal tract may be affected in up to 25% of patients with clinical symptoms suggestive of CMV infection [36, 37]. CMV infection may determine a progressive disruption of epithelial and mesenchymal cells, causing a progressive ulceration of the epithelial layer of small intestine and colon [38]. Colonoscopic findings in the four patients with CMV colitis in our study included microerosion and edematous mucosa with multiple erosions. CMV colitis may present in association with other IBDs, probably as a consequence of the immune dysregulation occurring in patients with CMV infection, and this results in more severe clinical course [8, 39, 40], as reported in our patient.

Histological findings included increased enterocyte apoptosis, which is caused by viral infection, so it is difficult to distinguish CMV colitis from GVHD on gastrointestinal biopsy, so that definitive diagnosis in kidney transplant patients requires histologic findings of characteristic inclusion bodies on haematoxylin and eosin staining [26, 39, 40].

The relatively small number of patients is the main limit of this study: however, the study included one of the largest series reported in literature evaluating the incidence of gastrointestinal complications during colonoscopy screening; moreover, this analysis regarded a homogeneous group of transplant recipients, allowing for the elimination of many confounding variables, such as race, and different immunosuppressive protocols. Not all eligible transplant patients underwent a diagnostic colonoscopy and this could underestimate the real prevalence of inflammatory disease; moreover, patients with low or moderate symptoms not affecting the social activities were usually not scheduled for diagnostic colonoscopy.

Despite these limitations, this study described the clinical and histological features of post-transplant colitis in kidney transplant population.

In conclusions, immunosuppression may predispose kidney transplant recipients to a chronic injury of colonic mucosa or to an increased rate of infections, finally resulting in a potentially high incidence of gastrointestinal complications. Fecal calprotectin and FIT are useful diagnostic tools for the diagnosis of inflammatory bowel disease, although they are not completely predictive of response to therapy. While most colitis may benefit from an immunosuppressive drug dosage reduction, *de novo* IBDs and CMV colitis may have a more severe course. Diagnostic colonoscopy should be encouraged in all transplant patients with refractory diarrhea and gastrointestinal symptoms, to allow a prompt diagnosis and a timely treatment, finally improving the quality of life and long-term outcomes of affected patients.

## MATERIALS AND METHODS

All kidney transplant recipients who underwent a diagnostic colonoscopy for any indication between 2014 and 2018 at the Organ Transplant Unit of the University Hospital of Catania were included in the study.

Kidney transplant recipients were eligible for this study if they had a negative pre-transplant colonoscopy, and had a functioning graft. Exclusion criteria were known or suspected familial colorectal cancer syndrome or known gastrointestinal disease.

Patients were evaluated for gastrointestinal complications and post-transplant colitis. Post-transplant colitis was defined as the presence of clinical symptoms (diarrhea, abdominal pain, rectal bleeding), with compatible histological features of IBD, MMF/MPA-related colitis and CMV colitis. Patients with normal colonoscopic findings were used as controls.

Post-transplant colitis were defined according to their histological features [2, 3, 9, 26]: MMF/MPA-colitis was defined by the presence of gastrointestinal symptoms not otherwise related to any other etiology, with endoscopic and histological features suggesting MMF/MPA-colitis, including Cryptic Cell apoptosis, atrophy of the crypt, cryptic abscesses with eosinophils infiltrate [2, 3, 26, 25]. IBD-like pattern was defined by the presence of patchy colitis, pancolitis, ileitis with multiple ulcers at colonoscopy with histological features of cryptitis and crypt destruction [2, 3, 25]. CMV-colitis pattern was defined by the presence of immunohistochemical stains positivity in association with cytopatic changes [40, 41]. All patients presenting with diarrhea had stool studies before colonoscopy to exclude *Clostridium difficile, Giardia, Cryptosporidium, Shigella, Salmonella*, and *Escherichia coli* infections.

Study participants completed a two faecal immunochemical tests (FIT) for human haemoglobin before colonoscopy. Moreover, fecal calprotectin (FCP) was measured using the quantitative enzyme-linked immunosorbent assay Quantum Blue Calprotectin Extended (Buhlmann, Basel, Switzerland).

Recorded variables included demographics, donor age, cause of end-stage renal disease, indication for colonoscopy (screening for colorectal cancer, diarrhea, gastrointestinal bleeding, abdominal pain), time on dialysis, waiting time, time since transplantation, histological and endoscopic features of each patient (normal colonoscopy, adenoma/polyps, diverticulosis, hyperemia, erosions/ulcers), FIT and FCP values. All colonic biopsies were examined by a single pathologist, experienced in transplantation and gastrointestinal pathology.

Kidney transplant recipients received a standard three-drug immunosuppressive therapy, with or without induction therapy with anti-interleukin-2 receptor antibodies (Simulect, Novartis, Basel, Switzerland) or with antithymocyte globulin (ATG-Fresenius, Fresenius, Bad Homburg, Germany), basing on both donor and recipient characteristics, as previously described [42].

### Statistical analysis

Results and patients characteristics are reported as raw values and percentages for categorical data and as mean **±** standard deviation **(**SD). Comparison of means and percentages between patients who developed colitis and patients with normal colonoscopic and histological findings was estimated by the unpaired two Student’s t-test or Mann-Whitney U test, as appropriate. p value < 0.05 was considered as statistically significant.
